# 1150. Pediatric Osteoarticular Infections Caused by Mycobacteria Tuberculosis Complex: A Twenty-Six Year Review of Cases in San Diego, California

**DOI:** 10.1093/ofid/ofab466.1343

**Published:** 2021-12-04

**Authors:** Ian Drobish, Nanda Ramchandar, Vanessa Raabe, Alice Pong, John S Bradley, Christopher R Cannavino

**Affiliations:** 1 University of California, San Diego, San Diego, California; 2 University of California San Diego, Carlsbad, California; 3 NYU Grossman School of Medicine, New York, New York; 4 University of California San Diego/Rady Children’s Hospital, San Diego, California; 5 University of California at San Diego, San Diego, California

## Abstract

**Background:**

Osteoarticular infections (OAI) account for 10-20% of extrapulmonary *Mycobacteria tuberculosis* (MTB) complex infections in children. Given the rarity of MTB OAI, the epidemiology, disease manifestations, and treatment are poorly characterized. We describe 21 children treated for MTB complex OAI over a 26-year period at a tertiary pediatric center in southern California.

**Methods:**

We conducted a retrospective review of children diagnosed with MTB complex OAI and cared for between 31 Dec 1992 to 31 Dec 2018 at a single tertiary care pediatric hospital with close proximity to the United States-Mexico border.

**Results:**

We identified 21 children with MTB complex OAI during the study period (Table 1). Concurrent pulmonary disease (4.8%), meningitis (9.5%), and intra-abdominal involvement (14.3%) were all observed. MTB complex was identified by culture from operative samples in 15/21 children (71.4%); 8/15 (51.3%) cultures were positive for *Mycobacterium bovis.* Of the eight cases of vertebral OAI (the most common site), one was culture-positive for *M. bovis*. Open bone biopsy was the most common procedure for procurement of a tissue sample and had the highest culture yield (Table 2). The median duration of antimicrobial therapy was 52 weeks (IQR 52-58). Successful completion of therapy was documented in 15 children (71.4%). Seven children (33.3%) experienced long term sequelae related to their infection.

Table 1. Twenty-one children with Mycobacteria tuberculosis complex osteoarticular infections.

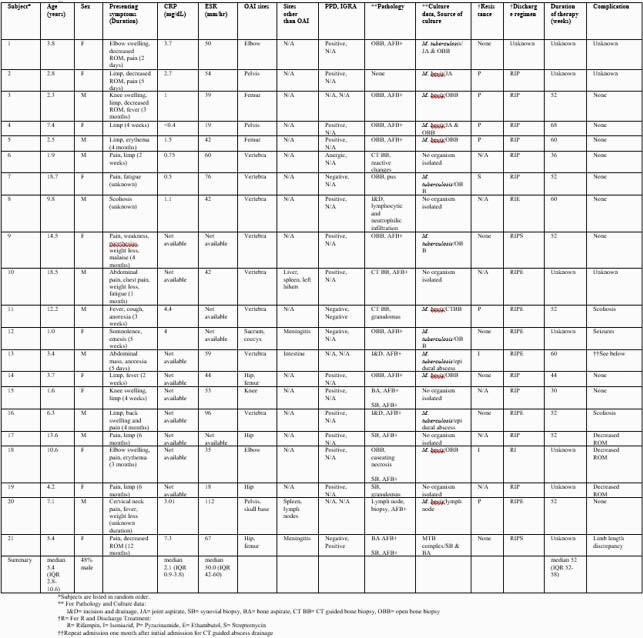

Table 2. Surgical sample type and percent positivity.

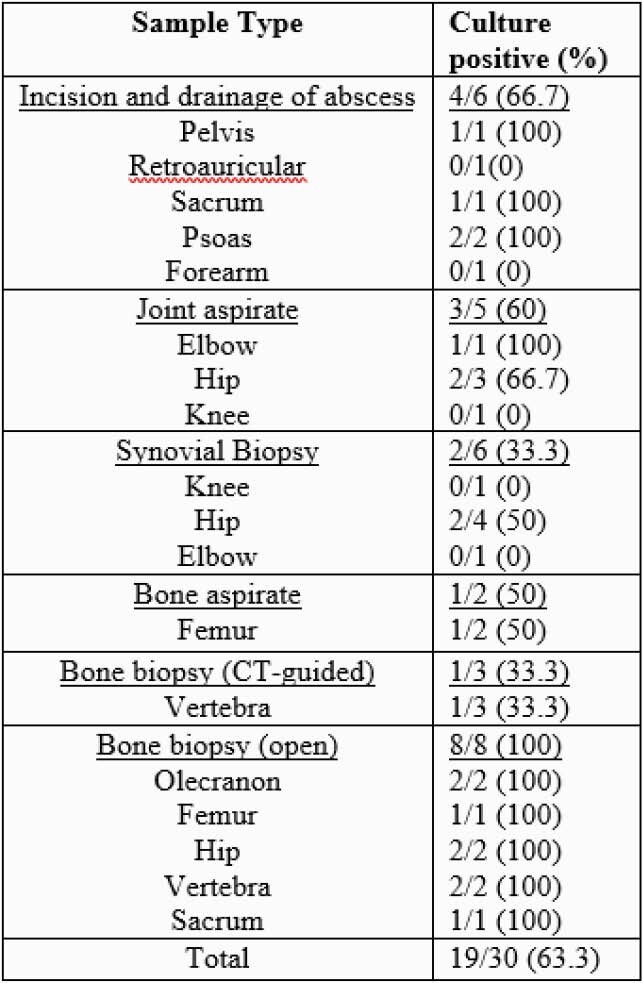

**Conclusion:**

Among the 21 children with MTB complex OAI assessed, 8 of 15 (53.3%) children with a positive tissue culture had *M. bovis* (intrinsically resistant to pyrazinamide), representing a higher percentage than in previous reports and potentially reflecting its presence in unpasteurized dairy products in the California-Baja region. Local epidemiological trends in endemic MTB complex species should be considered when evaluating and managing MTB complex OAI. Bone biopsy produced the highest culture yield in this study. Given the rarity of this disease, multicenter collaborative studies are needed to improve our understanding of the presentation and management of pediatric MTB complex OAI.

**Disclosures:**

**Vanessa Raabe, MD, MSc**, **Pfizer** (Scientific Research Study Investigator, Other Financial or Material Support, Editorial support)**Sanofi** (Scientific Research Study Investigator)

